# Streptomyces mayonensis sp. nov., isolated from the volcanic soils of Mt. Mayon, Philippines

**DOI:** 10.1099/ijsem.0.006900

**Published:** 2025-09-01

**Authors:** Gerald M. Aguilar, Kristel Mae P. Oliveros, Albert Remus R. Rosana, Rina B. Opulencia, Asuncion K. Raymundo

**Affiliations:** 1 University of the Philippines Los Baños, Laguna, Philippines; 2 Department of Science and Technology, Bicutan, Taguig, Philippines; 3 National Academy of Science and Technology, Taguig, Philippines

**Keywords:** new species, Mt. Mayon, Philippines, *Streptomyces mayonensis*, volcanic soils

## Abstract

A novel actinobacterium, designated as strain A1-08^T^, was isolated from the volcanic soils of Mt. Mayon, Philippines. Phylogenetic analysis of the 16S rRNA gene sequence revealed that strain A1-08^T^ belonged to the genus *Streptomyces* with sequence similarity with *Streptomyces djakartensis* NBRC 15409^T^ (99.02%), *Streptomyces tuirus* NBRC 15617^T^ (98.88%) and *Streptomyces mutabilis* NBRC 12800^T^ (98.81%). Polyphasic characterization demonstrated distinct morphological, cultural, physiological and biochemical features with unique lipid and fatty acid profiles and respiratory quinones. The major phospholipids of strain A1-08^T^ are diphosphatidylglycerol, phosphatidylethanolamine, glycophospholipid with mannose and/or galactose and unidentified lipids such as aminolipid and phospholipid. The major cellular fatty acids were anteiso-C_15 : 0_, C_16 : 0_, anteiso-C_17 : 0_, iso-C_16 : 0_, iso-C_15 : 0_ and C_16 : 1_ *ω*7c. The respiratory quinones of the strain include MK-9 (H6), MK-9 (H8), MK-10 (H6) and MK-10 (H8). Multilocus analysis using the concatenated sequences of *atpD*, *gyrB*, *recA*, *rpoB* and *trpB* showed that the strain formed a distinct branch in the genus *Streptomyces.* Potential genes for environmental stress response and adaptation were identified in the genome of strain A1-08^T^ and its closest relative strains. Whole-genome sequencing confirmed A1-08^T^ as a novel species within *Streptomyces* with 57.80% digital DNA–DNA hybridization and 93.03% average nt identity value with the closest type strain *Streptomyces olivaceus* NRRL B-3009^T^. The name *Streptomyces mayonensis* sp. nov., derived from the Mayon Volcano in the Philippines, is hereby proposed (type strain A1-08^T^ =JCM 36345, =DSM 118395).

**Impact Statement**

This study provides crucial taxonomic clarification of strain A1-08^T^ through a polyphasic approach, confirming its affiliation with the genus *Streptomyces*, a group widely recognized for its prolific production of bioactive compounds. By resolving the taxonomic status of this strain – previously shown to possess antibacterial and cytotoxic activities – the study lays the groundwork for its potential development in pharmaceutical and biotechnological applications. The validated classification enhances the understanding of *Streptomyces* diversity and supports future research into the discovery and exploitation of novel microbial metabolites. Moreover, the genetic adaptability of the strain to environmental stresses showed remarkable evidence that *Streptomyces* species can thrive and survive in one of the least explored and dynamic environments such as Mayon Volcano.

## Data Summary

The authors confirm that all supporting data, code and protocols have been provided within the article.

## Introduction

*Streptomyces,* a genus within the phylum *Actinomycetota*, comprises Gram-positive, spore-forming, filamentous bacteria with remarkably high G+C content (69–78 mol%) and linear and moderately large genomes ranging from 8 to 10 Mb [1]. They have widespread distribution in environments such as soil [2,5], plant endophytes [6,7], rhizospheres [8,9], marine waters and sediments [10,11] and extreme environment such as the deep-sea ecosystem [12,14] and arid environments [15]. Notably, *Streptomyces* species produce a wide array of industrially and pharmaceutically significant secondary metabolites accounting for 80% of the world’s antibiotics [10]. Around 5–10% of the genome of many species of *Streptomyces* is devoted to the production of bioactive metabolites that exhibit antibacterial, anticancer, antiparasitic, antifungal and immunosuppressant activities [11].

Previous studies in the Philippines have explored actinomycetes that produce novel and specialized metabolites, including the isolation of bioactive compound-producing actinomycetes from cave habitat [16], marine sediments [17,20] and urban green space [21]. However, volcanic habitats in the Philippines remain uninvestigated until 2021 when Oliveros *et al*. [22] explored Mt. Mayon, one of the most active volcanoes in the Philippines, for actinomycetes. During the investigation of actinomycetes with antimicrobial and cytotoxic activities from Mt. Mayon, strain A1-08^T^ was isolated from the volcanic soils collected 500 m above sea level. Strain A1-08^T^ showed antagonistic activity against *Salmonella enterica*, *Klebsiella pneumoniae*, *Staphylococcus aureus,* methicillin-resistant *S. aureus*, *Candida albicans*, *Aspergillus niger* and *Fusarium* sp. The strain also exhibited anticancer properties against human colorectal cancer (HCT116) cell line. This study reports the characterization, identification and taxonomic classification of strain A1-08^T^ through a polyphasic taxonomic approach.

## Methods

### Isolation and ecology

Strain A1-08^T^ was isolated from the volcanic soils of Mt. Mayon situated in Malilipot, Albay (13° 16′ 25.3″ N 123° 43′ 22.0″ E). Serial dilution was performed by suspending 10 g of the soil sample in 90 ml of 0.85% saline solution. A volume of 0.1 ml was aliquoted from 10^−4^ to 10^−6^ and spread plated on actinomycete isolation medium (grams per litre: 2.0 g sodium caseinate, 0.10 g l-asparagine, 4.0 g sodium propionate, 0.5 g dipotassium phosphate, 0.1 g magnesium sulphate, 0.001 g ferrous sulphate and 15.0 g agar) [23] supplemented with 100 ug ml^−1^ streptomycin and 100 units of nystatin. The inoculated plates were incubated at 37 °C for 10 days. Strain A1-08^T^ was purified by repeated streaking on yeast extract-malt extract (ISP 2) [24] agar plates. The purified strain was maintained in glycerol suspension (60% v/v) at −20 °C.

### Morphology

Phenotypic characteristics of strain A1-08^T^ were compared vis-à-vis with its closest relative type strains, *Streptomyces olivaceus* NRRL B-3009^T^ and *Streptomyces coelicoflavus* NBRC 15399^T^, based on the digital DNA–DNA hybridization (dDDH) results from Type Strain Genome Server (TYGS) database [25]. The cell morphology of strain A1-08^T^ was visualized using scanning electron microscopy (SEM). A 3 cm^2^ agar block of ISP 2 agar with sporulating strain A1-08^T^ was dehydrated using a 40 °C hot-air oven overnight [26] and subjected to SEM analysis conducted by the Department of Science and Technology-Advanced Device and Materials Testing Laboratory (DOST-ADMATEL), Taguig, Philippines. A representative of the sample was mounted on carbon conductive tape and sputter-coated with platinum prior to SEM analysis (Dual Beam Helios Nanolab 600i, FEI, USA).

The cultural characteristics of strain A1-08^T^ and its closely related strains were evaluated according to the guidelines of the International *Streptomyces* Project [25]. Strain A1-08^T^ was inoculated on tryptone-yeast extract agar (ISP 1), ISP 2, oatmeal agar (ISP 3), inorganic salt-starch agar (ISP 4), glycerol-asparagine agar (ISP 5), peptone-yeast extract iron agar (ISP 6), tyrosine agar (ISP 7), nutrient agar (NA) and tryptic soy agar (TSA) [24]. All agar plates were incubated at 28 °C for 14 days.

### Physiology and chemotaxonomy

The growth range of strain A1-08^T^ and its closely related type strains at various temperatures, pH levels and NaCl concentrations was determined. ISP 2 agar plates, in triplicate, were incubated at 5, 10, 15, 25, 30, 35 and 42 °C for 14 days [27]. To determine the pH growth range and optimum, the pH of ISP 2 broth was adjusted using the buffer system reported by Xu *et al*. [28]. Strain A1-08^T^ was inoculated to pH-adjusted ISP 2 broth, in duplicate, and incubated for 7 days with shaking (100 r.p.m., 28 °C). The growth range and optimum in various NaCl concentrations were determined [3] by supplementing NA with various NaCl concentrations (% w/v): 1, 3, 5, 7, 9, 11, 13 and 15. Agar plates were incubated at 30 °C for 14 days. The utilization of various carbon sources (glucose, fructose, xylose, sucrose, mannose, lactose, cellobiose and arabinose) was determined by inoculating buffered-washed cells of strain A1-08^T^ on carbon utilization medium as described by Shirling and Gottlieb [24]. To determine other biochemical and enzymatic characteristics of the strain, API^®^ CORYNE (bioMérieux, France) and API^®^ ZYM (bioMérieux) were used following the manufacturer’s protocol.

The chemotaxonomic analyses of polar lipids, fatty acid methyl esters (FAMEs) and respiratory quinones were performed by DSMZ (Braunschweig, Germany) from cultures grown for 3 days at 28 °C on TSA plates. Polar lipids were recovered from 200 mg freeze-dried cells with chloroform, methanol and 0.3% NaCl in water. Subsequently, the separation of the polar lipids was accomplished through two-dimensional silica gel chromatography, using chloroform-methanol-water as the first solvent and chloroform-methanol-acetic acid-water as the second solvent. Detection of total lipids and specific functional groups was achieved through various spray reagents as indicated by the DSMZ service [29]. FAMEs were collected from 300 mg wet biomass by saponification, methylation and extraction according to standard protocol [30]. The mixtures of FAMEs underwent separation through GC and were then identified using a flame ionization detection. In the following analysis, fatty acids were specifically identified through a GC-MS run performed on an Agilent GC-MS 7000D system [31]. Peaks were identified using retention time and mass spectra, and the naming of the fatty acids was performed according to the MIDI databases. Respiratory quinones were obtained from 500 mg wet biomass through hexane extraction and subsequent purification via a silica-based solid phase extraction. The purified samples were then subjected to HPLC with a reverse-phase column, and absorption spectra were recorded [31]. Relative quantification was performed at 270 nm for ubiquinones and 326 nm for menaquinones. Complex mixtures were analysed using UHPLC-ESI-qTOF system [32].

### 16S rRNA gene phylogeny

Genomic DNA was extracted from A1-08^T^ grown for 72 h in ISP 2 broth (28 °C, 28 ***g***) using Wizard genomic DNA purification kit (Promega, USA). The 16S rRNA gene was amplified in 0.2 ml thin-walled microfuge tube containing 1× Phusion GC buffer, 10 mM deoxynucleotide triphosphates mix, 3% DMSO, 10 µM each of primer pair, 1 U Phusion high-fidelity DNA polymerase (NEB, MA, USA) and 50 ng of genomic DNA, using the universal 16S rRNA gene primers 27F (5′ AGAGTTTGATCMTGGCTCAG 3′) and 1492R (5′ TACGGYTACCTTGTTACGACTT 3′) [33]. The amplification was performed using a Veriti thermal cycler (P/N 4375786, Life Technologies, USA) following these conditions: heated lid (98 °C); initial denaturation at 96 °C for 30 s; 25 cycles of denaturation (95 °C, 10 s), annealing (49 °C, 30 s) and extension (72 °C, 45 s); and final extension (72 °C, 10 min). The PCR amplicons were viewed through agarose gel electrophoresis (1% agarose, 0.5× tris-boric acid-ethylenediaminetetraacetic acid buffer), stained with ethidium bromide and visualized under ChemiDoc™ XRS Gel Documentation system (Bio-Rad, USA). QiaQuick PCR purification kit (Qiagen, Germany) and Qubit fluorometry v2.0 were used to column-purify and quantify the amplicons, respectively. The PCR products were sent to Macrogen Inc., Korea, for sequencing using the BigDye Terminator v3.1 cycle sequencing kit in an ABI 3730 DNA Analyzer (Applied Biosystems, USA). An internal primer 907R was used in addition to 27F and 1492R primers for 16S rRNA gene [33]. The 16S rRNA gene sequence was deposited in the NCBI database with accession number MN121123. A phylogenetic tree based on 16S rRNA gene sequence was generated using the Type (Strain) Genome Server [25]. Meanwhile, the closest neighbours of strain A1-08^T^ were queried using EzBioCloud [34].

### Genome features

Strain A1-08^T^ was grown in tryptic soy broth (Difco Laboratories, USA) at 28 °C for 96 h with shaking (28 ***g***). Extraction protocols of the genomic DNA and sequencing, cited in Oliveros *et al*. [22], were followed from the methods described by Daas *et al*. [35]. Biomass was collected using a 0.22 µm Stericup filtration unit (Millipore Sigma, USA), and the cells were washed with ice-cold 1× Tris EDTA (100 mM Tris-HCl, 50 mM EDTA and pH 8.0) buffer. Ten grams of the cell biomass were transferred to a microcentrifuge tube and resuspended with 100 µl 100 mM Tris-HCl buffer pH 8.0. Achromopeptidase (5 mg)-lysozyme (10 mg) combination (Sigma, St. Louis, MO, USA) was used to pre-treat the cell suspension and then incubated at 37 °C for 20 min. After the incubation period, 2 mg ml^−1^ of proteinase K (Qiagen) was added to the suspension (56 °C, 30 min) followed by the addition of 0.5 mg ml^−1^ DNase-free RNase A (37 °C, 30 min) (Thermo Fisher Scientific, USA). The genomic DNA of the pre-treated slurry was extracted using DNeasy Blood and Tissue DNA extraction kit (Qiagen) according to the manufacturer’s protocol. The quality and quantity of extracted genomic DNA were assessed using NanoDrop spectrophotometry, Qubit fluorometry and microfluidics-electrophoresis (BioAnalyzer, Agilent, USA). A Nextera XT DNA sequencing library (1 ng) was prepared, and sequencing was performed using NextSeq kit (2×150 bp).

For genome assembly and annotation, default parameters of the software were used unless otherwise stated. FastQC v0.11.8 by Andrews [36] was used to read the quality of the genome sequence. BBDuk v38.35 [37] was used to adapter-trim the raw reads, using the following parameters ktrim, r; k, 23; mink, 11; hdist, 1; tpe; tbo; minlen, 100; trimq, 10; and ref, adapters. BBDuk v38.35 was also used to filter the trimmed reads using the following parameters: k, 31; ref; artefacts; and phix. Reads were error-corrected using BBDuk v38.35 (with the parameters ecc, t; keepall; passes, 1; bits, 16; and prefilter). Further trimming was performed by quality scores using BBDuk v38.35 with the following parameters: qtrim, r; trimq, 10; and minlen, 100. The resulting short paired-end reads (corrected to 100–150 bp per read) were *de novo* assembled into contigs using SPADES v3.11.1 with k=21, 33, 55 and up to 77. The evaluated assemblies were subjected to Quast v3.1 CG view software and were used to construct the genome map, while Prokka was used in genome annotation.

The draft genome was submitted to the Microbial Genome Atlas (MiGA) [38] and queried under the TypeMat database. The resulting information gave classification to compare with the related genomes based on average nt identity (ANI) and aa identity values. A phylogenetic tree was generated using the Type (Strain) Genome Server [25]. Pairwise comparisons were conducted through Genome blast Distance Phylogeny (GBDP) approach, and accurate intergenomic distances using the algorithm ‘trimming’ and distance formula d_5_. The draft genome sequence of strain A1-08^T^ was deposited in the NCBI GenBank database with the accession number JACBYP000000000.

### Genetic adaptability to environmental stresses

Physiologically important genes were assessed using the SEED analysis feature of the Rapid Annotations using Subsystems Technology (RAST). The whole-genome sequences of strain A1-08^T^ and its closest relative strains were downloaded from the NCBI and submitted to RAST for genome annotation. Genes associated with stress response and environmental adaptation were mined from the genomes and related in comparison to previously published studies.

### Multilocus sequence analysis

The multilocus sequence analysis (MLST) genes of strain A1-08^T^ (*atpD*, *gyrB*, *rpoB*, *recA* and *trpB*) were accessed through the SEED viewer interface of RAST [39] and the ‘features in subsystems’ which provided all the genes present in the annotated whole-genome sequence. The housekeeping genes of the top 20 closely related type strains based on the Type (Strain) Genome Server results were collected from the NCBI database. All gene sequences were initially aligned, trimmed and concatenated in a head-to-tail manner (*atpD-gyrB-rpoB-recA-trpB*). The phylogenetic trees of the concatenated gene were reconstructed using maximum-likelihood, maximum-parsimony and neighbour-joining algorithms using mega 11.0.13 [40]. The MLST evolutionary distances were computed using Kimura’s two-parameter model in mega 11.0.13.

## Results and discussion

### 16S rRNA gene phylogeny

**Fig. 1.**
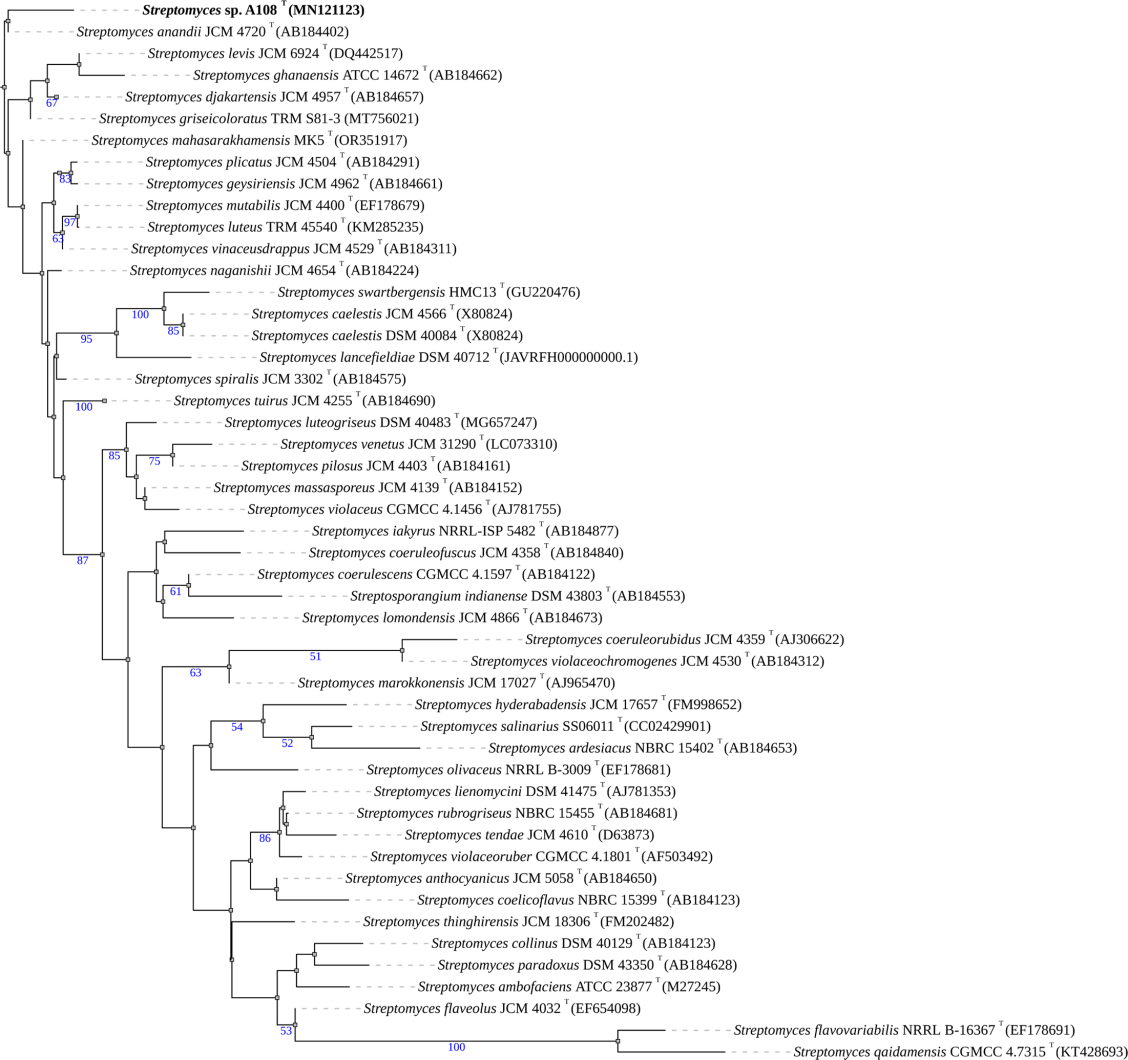
The phylogenetic tree was constructed using FastME v2.1.6.1, based on GBDP distances derived from 16S rDNA gene sequences. Branch lengths are represented according to the GBDP distance formula d5. Bootstrap support values greater than 50%, obtained from 100 GBDP pseudo-replications, are indicated above the branches. The average branch support was 46.7%. The tree was midpoint-rooted.

The partial 16S rRNA gene sequence of strain A1-08^T^ was aligned (1,442 bp) with the type strains of validly published *Streptomyces* species, available in the database of EzBioCloud. The strain A1-08^T^ shared the highest gene sequence similarity with *Streptomyces djakartensis* NBRC 15409^T^ (99.02%), *Streptomyces tuirus* NBRC 15617^T^ (98.88%) and *Streptomyces mutabilis* NBRC 12800^T^ (98.81%), where values were all above the threshold of 98.65–98.7% for prokaryotic species delineation [41]. Fig. 1 shows that strain A1-08^T^ was placed in a poorly supported distinct node loosely associated with *Streptomyces anandii* JCM 4720^T^ (98.5%) and distant from its closest relative strains.

### Genome-based phylogeny and comparative genomic analysis

**Fig. 2.**
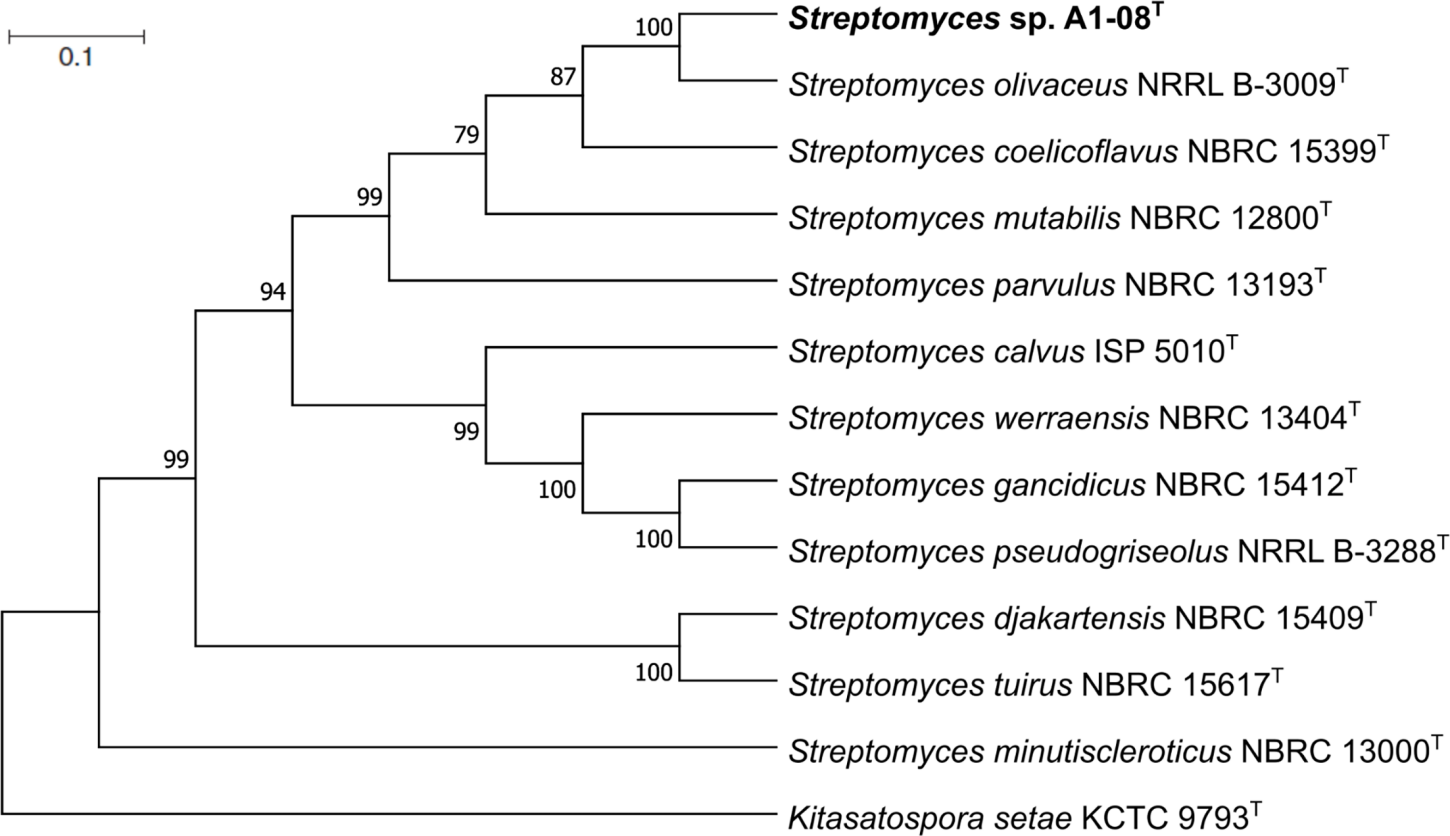
Maximum-likelihood tree based on MLSA of the concatenated partial sequences of five housekeeping gene sequences (*atpD*, *gyrB*, *recA*, *rpoB* and *trpB*) showing phylogenetic relationship of strain A1-08 and its closely related *Streptomyces* type species. Only bootstrap values above 50% (percentages of 1,000 replications/iterations) are displayed. Asterisks indicate branches that can be recovered in both neighbour-joining and maximum-parsimony trees. *Kitasatospora setae* KCTC 9793^T^ was used as an outgroup. Bar 0.1 indicates nt substitution per site.

dDDH and ANI were performed to elucidate the correct taxonomic species rank of strain A1-08^T^. The 16S rRNA gene sequence obtained from the whole-genome sequence of strain A1-08^T^ was identical to the sequence amplified through PCR. The dDDH and ANI values between the whole-genome sequence of strain A1-08^T^ and its closely related strain *S. olivaceus* NRRL B-3009^T^ were 57.8% and 93.0%, respectively. These values supported the novelty of the strain given the phylogenetic definition of species with dDDH <70% and ANI <95% [42,43]. MLST of selected housekeeping genes (*atpD*, *gyrB*, *recA*, *rpoB* and *trpB*) also confirmed that strain A1-08^T^ formed a separate clade from *S. olivaceus* NRRL B-3009^T^, which was consistent with the phylogenetic tree based on the whole-genome sequences of related taxa (Figs2  3, respectively). Pairwise distance calculations for A1-08^T^ with its related type strains exceeded the 0.007 threshold (Fig. 2) for *Streptomyces* species determination (Fig. 2) [44]. All these results confirm that strain A1-08^T^ represents a novel genomospecies within the genus *Streptomyces*.

**Fig. 3.**
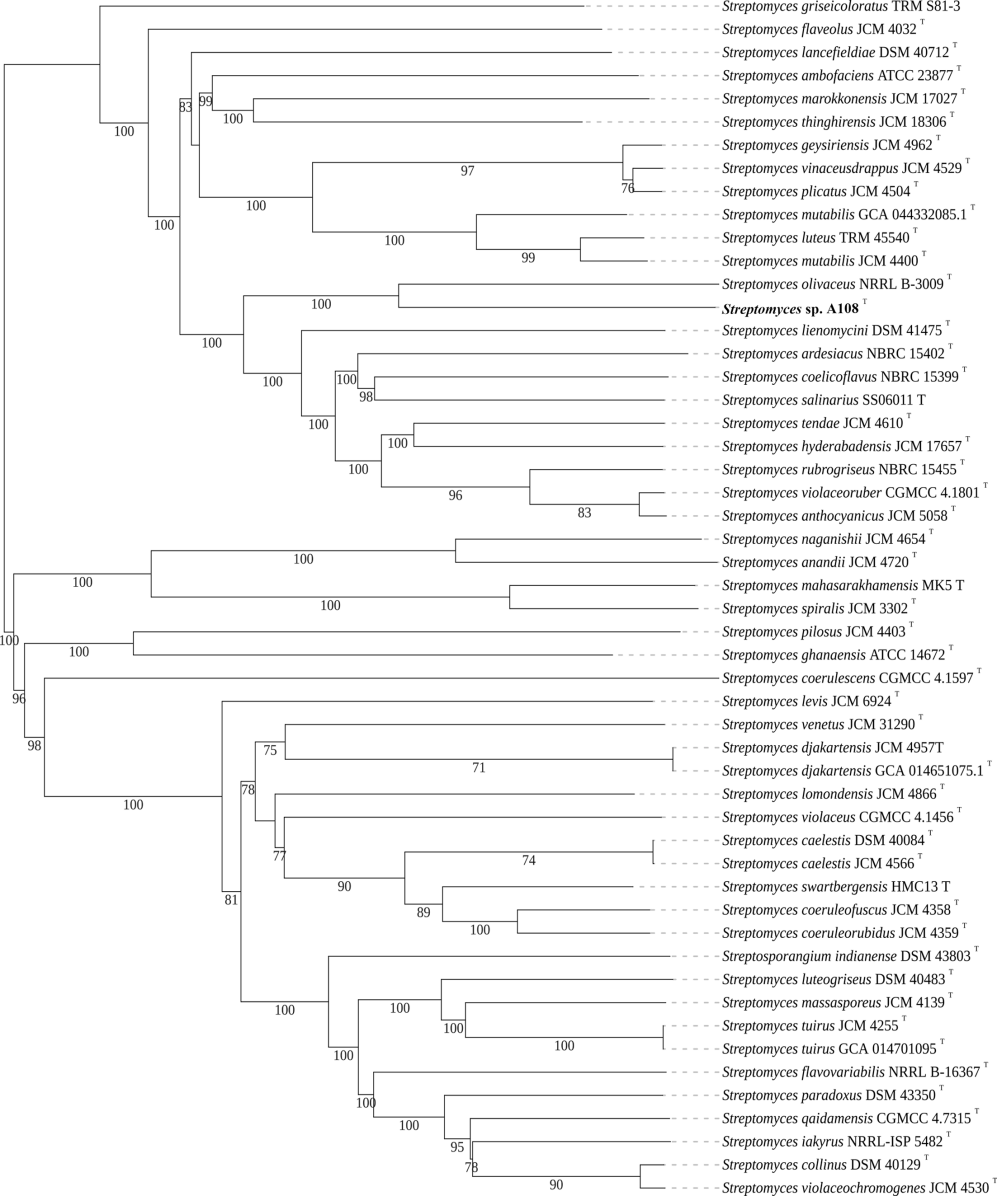
Phylogenomic tree based on the whole-genome sequence of strain A1-08^T^ and related type strains, inferred with FASTME v2.1.6.1 from GBDP distances calculated from genome sequences. The branch lengths are scaled in terms of GBDP distance formula d_5_. The number below the branches is GBDP bootstrap values greater than 50% from 100 replications. Accession numbers of the genome sequences are placed in brackets.

Isolate A1-08^T^ and its close relative strain *S. olivaceus* NRRL B-3009^T^ had a genome size of 8.85 and 8.58 Mb, G+C content of 72.2 and 72.4 mol%, N_50_ of 57,920 and 199,385, number of coding sequences 7,960 and 7,863 and RNAs of 68 and 77, respectively. The genomic characteristics of the strain exhibited high concordance with the taxonomic and molecular signatures defining the genus *Streptomyces*.

### Genomic analysis in relation to environmental stress and adaptation

One of the most ubiquitous environmental stresses in soil is osmotic stress. Due to drastic changes in the environment, soil bacteria such as *Streptomyces* species should develop osmoregulation mechanisms. These mechanisms involve both active (e.g. transport and synthesis of compatible solutes such as glycine betaine, proline and ectoine) and passive approaches (e.g. mechanosensitive channels) [45]. Strain A1-08 and its closely related type strains possessed genes for choline and betaine uptake and biosynthesis, encoded by *opuAA* (glycine betaine transport ATP-binding protein), *opuAB* (glycine betaine transport system permease protein) and *opuD* (glycine betaine transporter) and glycine facilitator protein. These genes act as osmoprotectants that transport compatible solutes into cells to help tolerate osmotic stress [46]. A complete biosynthetic gene cluster (BGC) for ectoine biosynthesis was also previously reported in strain A1-08^T^ [22].

Oxidative stress is also an environmental stress that occurs when there is excessive production of reactive oxygen species (ROS) that adversely affect various macromolecules (e.g. nucleic acids, proteins and lipids) in the cell. ROS accumulates due to accidental oxidation of flavins in the cell, which play an important role in aerobic metabolism of the organism, or due to the exposure of the micro-organism to extracellular sources of ROS [47]. Responses to oxidative stress involve the participation of various enzymes like superoxide dismutase, peroxidase and catalase. The strain A1-08^T^ and its closely related type strain possess *soxR*, which encodes the SoxR protein that induces the expression of *soxS*, which in turn activates the transcription of other genes involved in antioxidant defence [48]. Moreover, *ahpC* that codes for alkyl hydroperoxide reductase, which is important for hydrogen peroxide detoxification, is present in all strains of *Streptomyces.* Other potential players for oxidative stress response by the *Streptomyces* strains in this study include *nsrR* gene (nitrite-sensitive transcriptional repressor) and *ohrR* gene (organic hydroperoxide resistance transcriptional regulator). The *fnr* (transcriptional regulator crp/fnr family), *cat* (catalase) and *sod* (superoxide dismutase) genes were recovered from all strains studied except for strain A1-08^T^.

SEED analysis revealed that all the *Streptomyces* strains possessed genes that code for degradation enzymes such as *chiC* (chitinase) and *aglA* (α-glucosidase), which are some of the important enzymes for soil bacteria [49]. *Streptomyces* also produce extracellular chitinase, which hydrolyses the β-1,4-glycosidic bond of chitin, an insoluble polymer consisting of repeating units of *N-*acetylglucosamine (GlcNAc), to generate GlcNAc that can serve as a carbon source [50]. Meanwhile, *α*-glucosidase or maltase hydrolyses the non-reducing end of α-1,4-linked glucose residues from an oligosaccharide [51].

Furthermore, genes for phytohormone production were also noted in all strains, such as *trpD*, which codes for indole-3-glycerol phosphate synthase, which is involved in the biosynthesis of an intermediate in the tryptophan biosynthetic pathway associated with the production of indole-3-acetic acid (IAA) that promotes plant growth [52]. Also found in the genome of all strains is anthranilate synthase (*trpAa*), which is involved in the first step and the rate-limiting enzyme in the biosynthetic pathway for tryptophan biosynthesis, which also leads to the production of IAA [53].

Various siderophores were seen in all strains. Siderophores scavenge iron from the environment and may serve as a virulence factor for pathogenic bacteria [54]. Genes for ferric hydroxamate ABC transporter substrate-binding protein (*fuhD*), ferric hydroxamate ABC transporter ATP-binding protein (*fuhC*) and ferric hydroxamate ABC transporter permease (*fuhB*) were recovered from all *Streptomyces* strains. Although genes for desferrioxamine biosynthesis and transport were not observed in the genome of strain A1-08^T^, two uncharacterized BGCs were coding for novel mirubactin [22]. Hydroxamic acids constitute a significant group of compounds, primarily valued for their ability to chelate metals. Micro-organisms, including pathogenic bacteria, employ hydroxamate-containing substances like siderophores to obtain iron [55]. On the other hand, desferrioxamine is a bacterial siderophore usually produced by *Streptomyces* species, which was found to promote the growth of some bacteria such as *Microbacterium*, and violacein production and pilus formation in *Janthinobacterium* [56]. Siderophores are said to be important in the survival of *Streptomyces* species in the soil. Iron sequestration of *Streptomyces* through the production of siderophores reduced the survival of other competing soil bacteria through iron starvation [57].

### Morphology

**Fig. 4.**
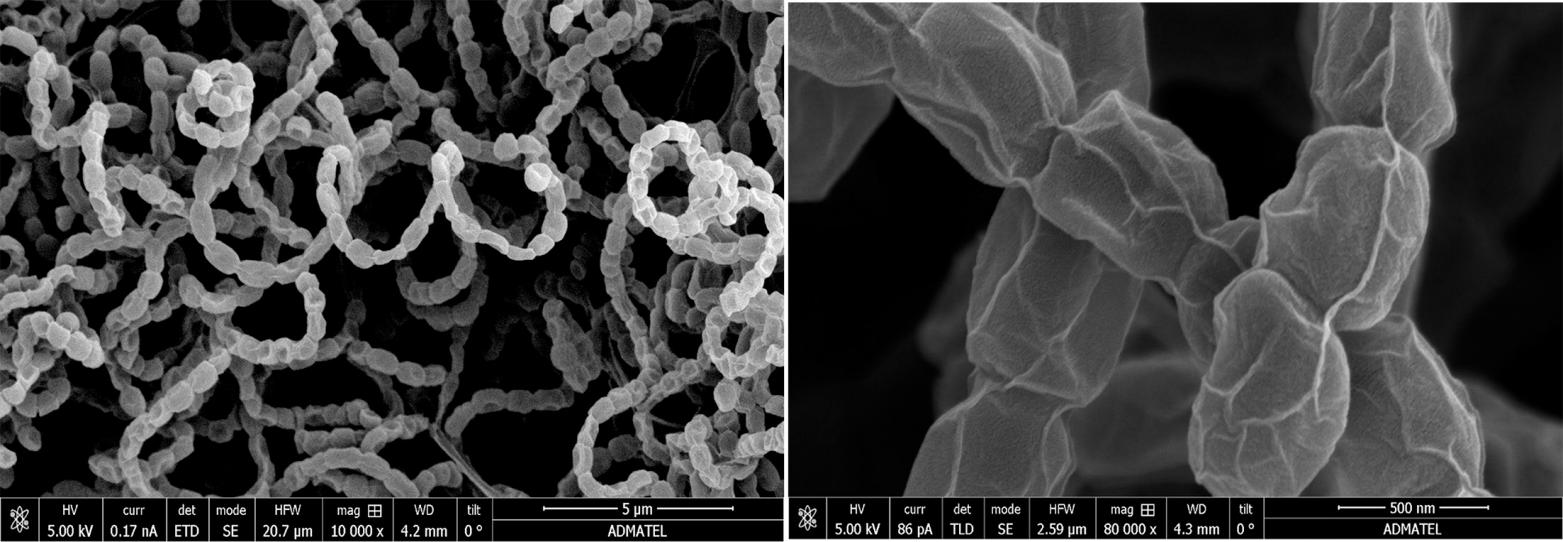
Scanning electron micrograph showing the spore chain morphology and spore surface of strain A1-08^T^ under 10,000× and 80,000× magnification, respectively.

Strain A1-08^T^ exhibited typical morphological features of the genus *Streptomyces*, aligning with Shirling and Gottlieb’s classification of *Streptomyces* species [26]. Strain A1-08^T^ exhibited a spiral spore chain similar to its closely related neighbour, *S. coelicoflavus* NBRC 15399^T^ [58,59]. SEM revealed that the strain produced spores with a rugose surface, measuring 637–790 nm in length and 447–578 nm in width (Fig. 4). Strain A1-08^T^ produced white to light grey aerial mycelia on ISP 1–7, NA and TSA after 2-week incubation. The substrate mycelia of the strain ranged from yellowish brown to brown. Brown diffusible pigment was observed on ISP 2 and ISP 7 after prolonged incubation of up to 21 days. No optically visible melanin production was observed on ISP 7. The detailed cultural characteristics of the strain A1-08^T^ are summarized in Table 1.

**Table 1.** Cultural characteristics of strain A1-08^T^ and its closely related strains on various culture media

Culture medium	A1-08^T^		*S. olivaceus* NRRL B-3009^T^		*S. coelicoflavus* NBRC 15399**^T^**	
	Aerial	Substrate	Aerial	Substrate	Aerial	Substrate
ISP 1	Light grey	Dark brown	Light grey	Dark brown	White	Dark brown
ISP 2	Light grey	Yellow brown	Light grey	Dark brown	White	Dark brown
ISP 3	White	Yellow brown	White	Black	Light grey	Beige
ISP 4	White	Brown	Light grey	Black	Light grey	Black
ISP 5	White	Dark brown	Light grey	Black	White	Beige
ISP 6	White	Dark brown	White	Beige	White	Beige
ISP 7	White	Beige	Light grey	Dark brown	White	Beige
nutrient agar	Light grey	Yellow brown	Light grey	Dark brown	White	Beige
TSA	White	Yellow brown	na	Beige	White	Beige

### Physiology and chemotaxonomy

Strain A1-08^T^ exhibited moderate growth at 25–37 °C and optimal growth at 30–35 °C. No growth was noted between 5 and 15 °C as opposed to its closest relative strains that had growth at 10 °C. Soil streptomycetes are regarded as mesophiles, thriving in moderate temperatures ranging from 10 to 37 °C [60]. Similar to A1-08^T^ that was isolated from the volcanic soils, a mesophilic growth range was observed in a novel soil actinomycete species isolated from a lava tube in Canary Island, Spain [61].

Isolate A1-08^T^ can be distinguished from its closely related neighbours, *S. olivaceus* NRRL B-3009^T^ and *S. coelicoflavus* NBRC 15399^T^, by its ability to grow at pH 10.0. *S. olivaceus* NRRL B-3009^T^, on the other hand, had growth at pH 4.0. *Streptomyces* are generally neutrophiles, growing between pH 6.0 and 8.0, with an optimum pH close to 7.0. Exceptions are some acidophilic streptomycetes that can grow between pH 3.5 and 6.5 with optimum pH of 4.5–5.5 and alkaliphilic actinomycetes that grow at 8.0–11.5 with optimum pH of 9.0–9.5 [24]. Acidophilic streptomycetes have been isolated from acidic and rhizospheric soils [28,62], while an alkaliphilic streptomycete was isolated from an alkaline escarpment region [63].

Strain A1-08^T^ demonstrated notable halotolerance, distinguishing it from closely related species by its ability to grow in the presence of 9.0% NaCl. Similar halotolerant streptomycetes have also been isolated from the environment, such as *Streptomyces chilikensis* that was isolated from the sediment of an estuarine coastal brackish water lagoon in India and could tolerate NaCl concentrations up to 12% [59]. A rhizospheric soil streptomycete, *Streptomyces triticagri*, also exhibited high tolerance to NaCl at up to 13% [64].

The isolate A1-08^T^ could be differentiated from its closest relative strains by being able to utilize xylose as the sole carbon source. Notably, *S. olivaceus* NRRL B-3009^T^ demonstrated utilization of ribose, while arabinose and maltose for *S. coelicoflavus* NBRC 15399^T^. Strain A1-08ᵀ also had positive reaction for nitrate reductase, while phylogenetic neighbors were negative. Strain A1-08ᵀ and *S. coelicoflavus* NBRC 15399^T^ had positive reaction for esterase. Furthermore, *S. coelicoflavus* NBRC 15399^T^ had positive reactions in urease, *β*-*N*-acetylglucosaminidase and leucine arylamidase. Both close neighbours had positive reactions in *α*-glucosidase and aesculin. The summary of the physiological and biochemical characteristics of strain A1-08^T^ and its related species is shown in Table 2.

**Table 2.** Differences in physiological characteristics, enzymatic activities and utilization of carbon sources for strain A1-08^T^ and its related taxa

Characteristic	A1-08^T^	*S. olivaceus* NRRL B-3009^T^	*S. coelicoflavus* NBRC 15399^T^
**Physiological** NaCl growth range (%)	0 to 9	0 to 7	0 to 7
Growth temperature (°C)	25 to 37	10 to 37	10 to 37
pH growth range	5 to 10	4 to 9	5 to 9
**Enzymatic activities** Urease	−	−	+
Esterase	+	−	+
Nitrate reductase	+	−	−
*α*-Glucosidase	−	+	+
*β*-*N*-acetylglucosaminidase	−	−	+
Aesculin	−	+	+
Leucine arylamidase	−	−	+
**Carbon utilization** Xylose	+	−	−
Arabinose	−	−	+
Ribose	−	+	−
Maltose	−	−	+

+ positive reaction

− negative reaction

**Fig. 5.**
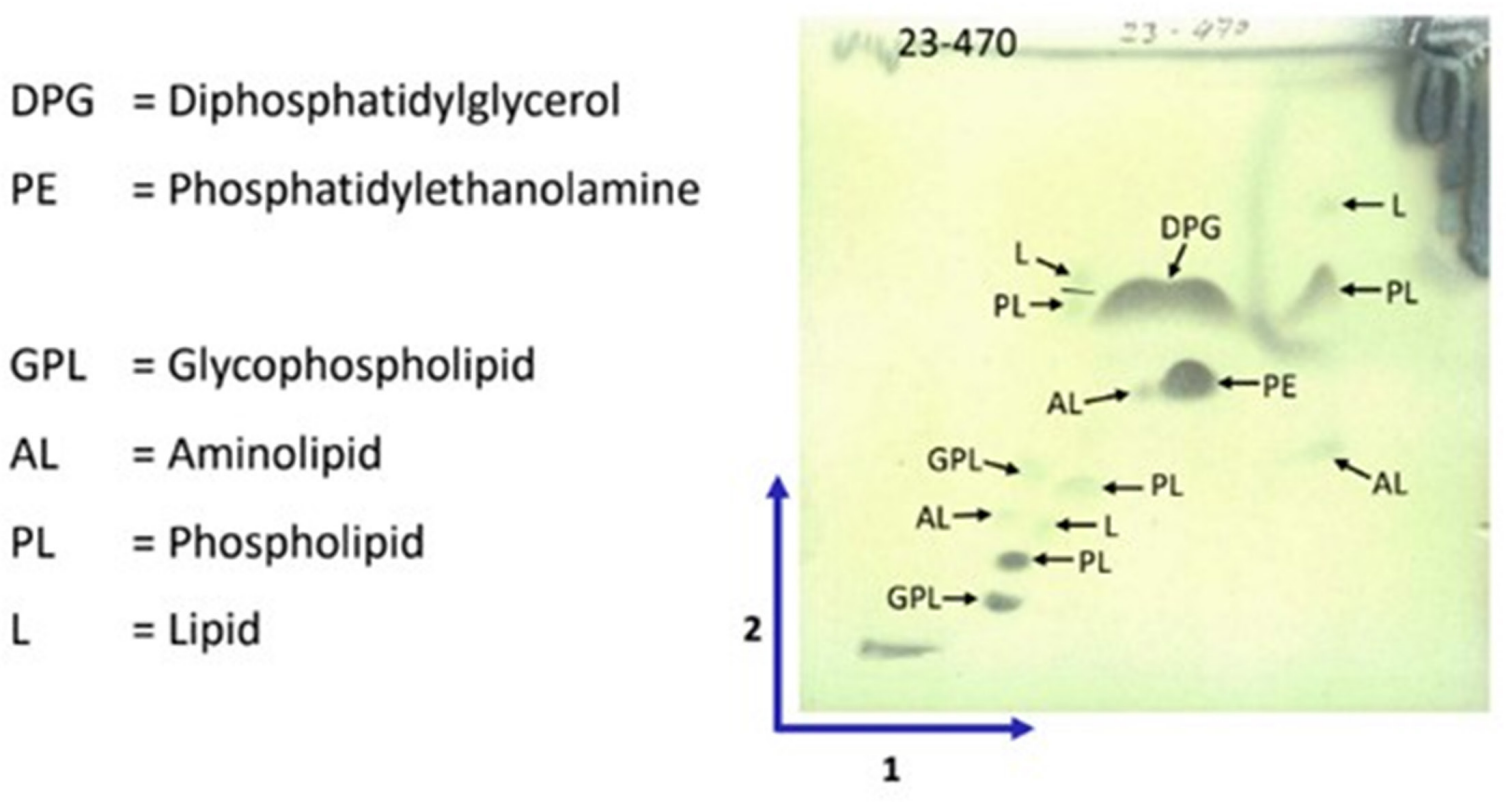
Chromatogram showing the polar lipids of *Streptomyces* sp. A1-08^T^ using two-dimensional silica gel TLC carried out by DSMZ.

Strain A1-08^T^ contained MK-9 (H6) (11.5%), MK-9 (H8) (84.8%), MK-10 (H6) (1.9%) and MK-10 (H8) (1.85%) as its respiratory quinones. The major cellular fatty acids (>5 %) were iso-C_15 : 0_ (7.0%), anteiso-C_15 : 0_ (28.5%), iso-C_16 : 0_ (9.8%), C_16 : 0_ (20.3%) and anteiso-C_17 : 0_ (15.4%). The polar lipids of the strain consisted of diphosphatidylglycerol (DPG), phosphatidylethanolamine (PE), glycophospholipid (GPL) (with mannose and/or galactose) and unidentified aminolipid (AL), phospholipid (PL) and lipid (L) (Fig. 5). These chemotaxonomic characteristics observed in strain A1-08^T^ were consistent with patterns of known species of the genus *Streptomyces* [58].

## Description of *Streptomyces mayonensis* sp. nov.

*Streptomyces mayonensis* (ma.yo.nen’sis, N.L. masc. adj. mayonensis, referring to the Mayon Volcano in the Philippines, the isolation source).

Aerobic, Gram-positive actinomycete that forms well-developed, branched substrate and aerial mycelia that differentiate into spiral spore chains comprising cylindrical rugose spores (0.64–0.67 µm×0.45–0.58 µm). Exhibits good growth on ISP 1 to 3, 6 and 7, NA and TSA, and sparse and moderate growth on ISP 4 and ISP 5, respectively; produces brown diffusible pigment on ISP 2 and ISP 7. Light grey to white aerial mycelia are formed on ISP 1 to 7 agar, NA and TSA. Substrate mycelia on ISP 1 and 5 agar media are dark brown, brown on ISP 4 agar and yellowish brown on ISP 2 and 3 agar, NA and TSA. Grows at 25–37 °C, pH 5–10 (optimum pH 6) and NaCl% up to 9%. Utilizes xylose, fructose, lactose, cellobiose, mannose and ribose, but not sucrose, mannitol, maltose and glycogen. Positive for nitrate reductase, esterase (C4), esterase lipase (C8), leucine arylamidase, acid phosphatase, pyrrolidonyl arylamidase, alkaline phosphatase, *β*-galactosidase, gelatinase, naphthol-AS-BI-phosphohydrolase and catalase. Negative for pyrazinamidase, lipase, *β*-glucuronidase, *α*-glucosidase, *N*-acetyl-*β*-glucosaminidase, aesculin, urease, valine and cystine arylamidases, trypsin and *α*-chymotrypsin activities. The major polar lipids are DPG, PE, GPL with mannose and/or galactose and unidentified AL, PL and L. The predominant fatty acids are anteiso-C_15 : 0_, C_16 : 0_, anteiso-C_17 : 0_ and iso-C_16 : 0_, and the menaquinones are MK-9 (H6), MK-9 (H8), MK-10 (H6) and MK-10 (H8). Potentially carries genes associated with the transport systems for osmolytes and siderophores, breakdown of oligosaccharides and polysaccharides and plant growth-promoting hormones, chaperones and proteins.

## Abbreviations

AL: aminolipid
ANI: average nt identity
BGC: biosynthetic gene cluster
DPG: diphosphatidylglycerol
FAMEs: fatty acid methyl esters
GBDP: Genome blast Distance Phylogeny
GlcNAc: *N*-acetylglucosamine
GPL: glycophospholipid
IAA: indole-3-acetic acid
L: lipid
MLST: multilocus sequence analysis
NA: nutrient agar
PE: phosphatidylethanolamine
PL: phospholipid
RAST: Rapid Annotations using Subsystems Technology
ROS: reactive oxygen species
SEM: scanning electron microscopy
TSA: tryptic soy agar
